# Mechanical Thrombectomy Complicated by Vascular Mass Extraction: A Case of Inferior Vena Cava Arteriovenous Hemangioma

**DOI:** 10.7759/cureus.83840

**Published:** 2025-05-10

**Authors:** Abshar Khan, Aaryan Patel, Neerav B Patel, Abbas Merchant, Minh-Tri Hoang

**Affiliations:** 1 Radiology, Lake Erie College of Osteopathic Medicine, Erie, USA; 2 Physical Medicine and Rehabilitation, Lake Erie College of Osteopathic Medicine, Erie, USA; 3 Internal Medicine, Lake Erie College of Osteopathic Medicine, Erie, USA; 4 Anesthesia, Lake Erie College of Osteopathic Medicine, Erie, USA; 5 Radiology, Rochester Regional Health, Rochester, USA

**Keywords:** arteriovenous hemangioma, inari clottriever, inferior vena cava, interventional radiology, intravenous malformation, neoplastic disease, radiology, thrombectomy, thrombolysis

## Abstract

We present a case of a 57-year-old female admitted for non-ST elevation myocardial infarction (NSTEMI), who was incidentally found to have a suspected inferior vena cava (IVC) thrombus. Transthoracic echocardiogram (TTE) demonstrated a right atrial lesion with suspected IVC involvement. Echocardiography incidentally reported a mass extending to the right atrium, and CT imaging reported possible IVC thrombus involvement. Abdominal ultrasound confirmed a suprarenal IVC thrombus. Interventional radiology (IR) performed mechanical thrombectomy and catheter-directed thrombolysis, which was complicated by a stuck catheter and unexpected retrieval of a vascular lesion. Histopathological analysis revealed the lesion to be an arteriovenous hemangioma (AVH). This case underscores the importance of considering rare vascular anomalies, such as AVH, in the differential diagnosis of IVC thrombus and highlights the diagnostic and procedural challenges in managing venous thromboembolism complicated by underlying vascular malformations.

## Introduction

Obstruction of the inferior vena cava (IVC) is an infrequent yet clinically significant condition that poses diagnostic and therapeutic challenges. While such obstructions are often attributed to thrombotic events, it is crucial to consider alternative etiologies, including neoplastic processes and vascular malformations, especially when imaging findings are atypical or when standard treatments prove ineffective [1]. Accurate identification of the underlying cause is essential, as management strategies differ substantially based on the specific diagnosis.

AVHs are uncommon vascular anomalies characterized by direct connections between arteries and veins, resulting in high-flow shunting. These lesions are typically located in the soft tissues of the head, neck, or extremities, with intravascular or visceral involvement being exceedingly rare [2,3]. Although rare, case reports have described similar vascular lesions in large central veins, including the superior vena cava [4]. The presence of an AVH within the IVC is particularly unusual and presents unique diagnostic and therapeutic considerations when compared to the more common forms of IVC occlusion. These include malignancy from nearby organs and thrombus.

This case report discusses a patient who presented with non-ST elevation myocardial infarction (NSTEMI) and was incidentally found to have an IVC lesion extending into the right atrium. Initially presumed to be thrombotic in nature, the lesion's resistance to both thrombolytic therapy and mechanical thrombectomy prompted further investigation, ultimately revealing a rare vascular anomaly. This case underscores the importance of maintaining a broad differential diagnosis when evaluating intravascular filling defects and highlights the complexities involved in managing such atypical presentations, particularly when procedural complications arise during catheter-based interventions [5].

## Case presentation

**Figure 1:**
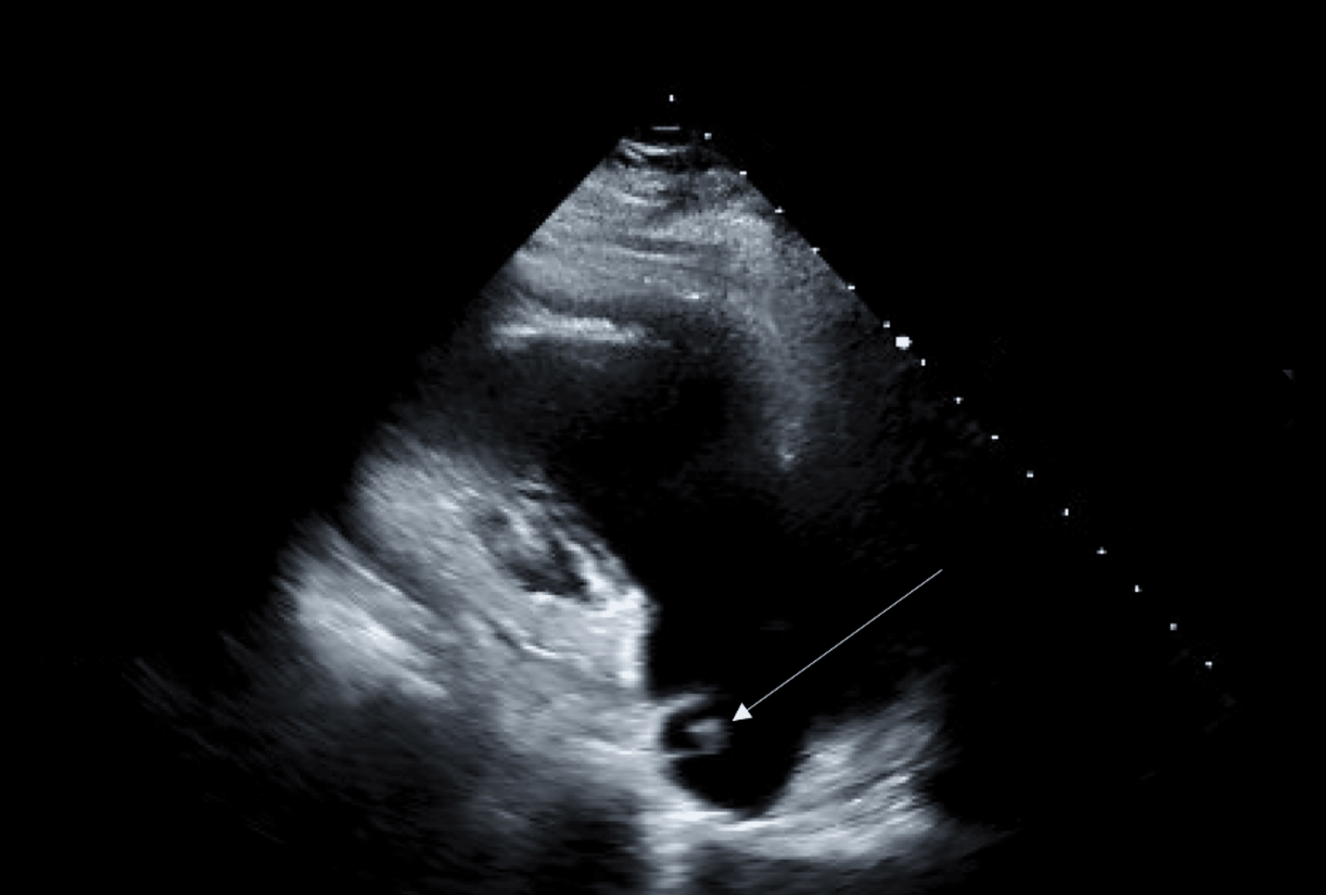
TTE showing a lesion in the right atrium (marked by arrow) TTE: transthoracic echocardiogram

A 57-year-old female with a past medical history of hypertension and remote intracranial hemorrhage presented to the emergency department with acute chest pain and tightness. On admission, she was found to have a hypertensive emergency with a blood pressure of 202/129 mmHg. Other vital signs were within normal limits. Initial management included aspirin, heparin, and nitroglycerin infusions. Pulmonary edema was noted on chest radiography and treated with furosemide. Laboratory results on admission revealed markedly elevated troponin I levels, consistent with NSTEMI (Table 1). TTE was planned to evaluate the patient's level of cardiac function (Figure 1).

**Table 1:** Cardiac markers upon admission HR: hour

Cardiac Markers	Patient Value	Reference Range
Troponin I, high sensitivity, 0HR	972 ng/L	0 - 15 ng/L [6]
Troponin I, high sensitivity, 1HR	4338 ng/L	0 - 15 ng/L [6]
Troponin I, high sensitivity, Delta 0-1HR	3366 ng/L	0 - 6 ng/L [7]

**Figure 2:**
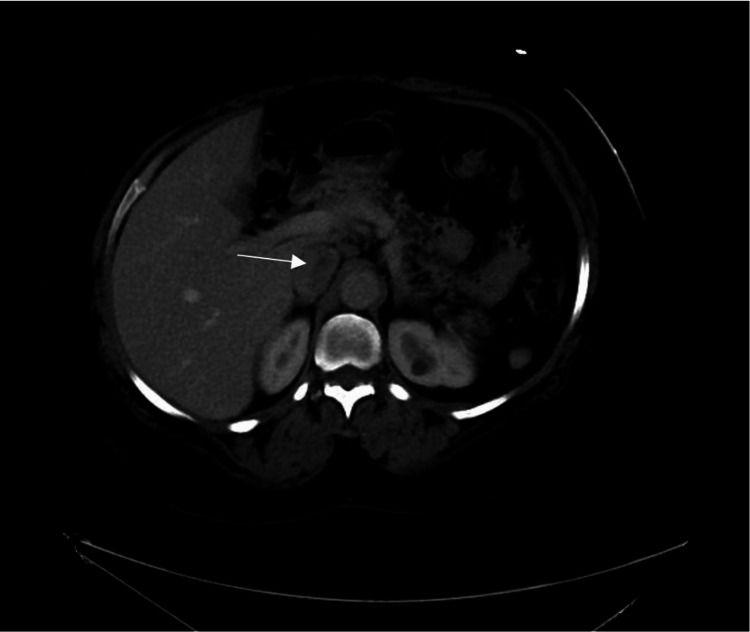
Abdominal CT scan with IV contrast demonstrating possible thrombus (marked by arrow)

CT with contrast of the chest, abdomen, and pelvis was inconclusive for definitive thrombus versus tumor. Radiology reported a well-defined linear filling defect within the left aspect of the intrahepatic and suprarenal IVC (Figure 2).

**Figure 3:**
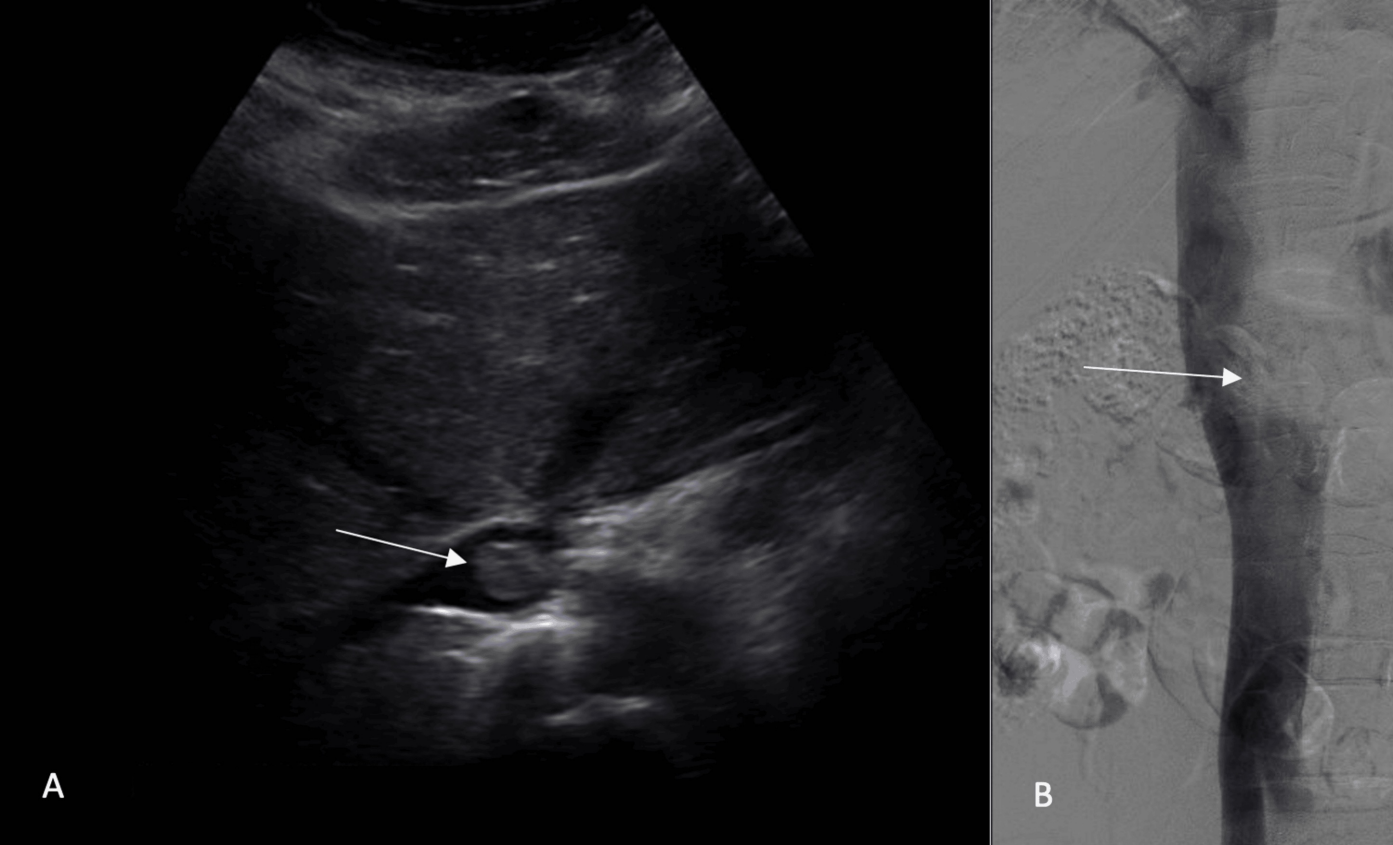
Imaging findings supporting the diagnosis of a lesion in the IVC A) Abdominal ultrasound demonstrating a nonocclusive echogenic structure (marked by the arrow) in the lumen of the upper abdominal IVC, B) Venocavogram showing a filling defect in the upper abdominal IVC (marked by the arrow) IVC: inferior vena cava

These findings prompted an abdominal ultrasound, which confirmed a non-occlusive echogenic structure in the suprarenal IVC, possibly consistent with thrombus (Figure 3A). A venogram with possible thrombectomy was then planned in order to assess the clot burden and plan for clot debridement (Figure 3B).

Initial attempts to mechanically retrieve the clot using the Inari FlowTriever device (Inari Medical, Irvine, CA, US) were unsuccessful. As an alternative approach, an infusion catheter was advanced to the suprarenal IVC, and alteplase, along with heparin, was administered via the sheath overnight in the ICU. After 12 hours, a repeat intervention was planned. The patient was preloaded with 300 mg of clopidogrel prior to IVC venography and repeat mechanical thrombectomy on hospital day four. Venography revealed a persistent filling defect in the hepatic segment of the IVC, indicating that alteplase therapy had failed to reduce the clot burden. This may have raised suspicion for an alternative diagnosis; however, this finding is not uncommon in chronic thrombi. The leading consideration was still thrombus, given its potentially life-threatening nature and the need for prompt anticoagulation or interventional management.

**Figure 4:**
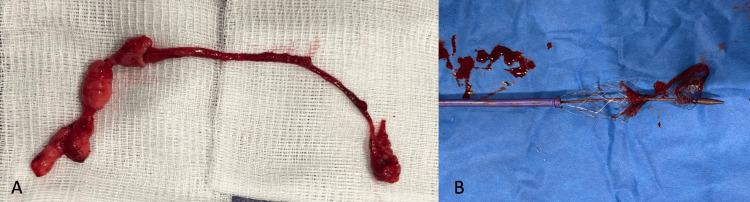
Key intraoperative findings in the thrombectomy procedure A) Elongated lobular lesion extracted from the suprarenal IVC measuring 18 cm in length and 1.3 cm in diameter, B) The ClotTriever catheter device retrieved following thrombectomy, showing significant deformation

While alternative etiologies, such as tumor thrombus or artifact, were considered, thrombus remains the most urgent and clinically significant diagnosis. With this consideration, mechanical thrombectomy was then attempted using the Inari ClotTrieverXL system, with access obtained via the right femoral vein. Multiple passes with the device failed to retrieve the thrombus, and the procedure was complicated by the catheter and coring element becoming trapped at the right groin access site. During an attempt to free the catheter with significant pulling force, an elongated, lobular lesion was unexpectedly retrieved (Figure 4A). Hemostasis was subsequently achieved using the Inari Flowstasis device. The lesion in the coring element was not compressible by the collapsing mesh and was unable to be advanced into the collection bag. The mechanical stress encountered during attempts to extract the thrombus resulted in significant deformation of the ClotTriever device (Figure 4b). The distorted structure underscores the complexity of the intervention and the challenges faced in achieving successful mechanical thrombectomy.

## Discussion

This case illustrates the diagnostic complexity of an IVC lesion initially suspected to be a thrombus but ultimately presumed to be an AVH. The initial imaging findings were inconclusive, necessitating a multimodal approach including echocardiography, CT, ultrasound, and venography. The lesion’s persistence despite thrombolysis and thrombectomy attempts raised suspicion for an alternative diagnosis; however, the presence of a filling defect and mass seen on abdominal ultrasound were findings consistent with thrombus as well, leading to an uncertain clinical diagnosis.

The final pathology report identified the mass as a benign vascular lesion consistent with that of an AVH, supported by immunohistochemical staining with desmin, CD34, pan cytokeratin, PAX8, and HMB45. AVH is a rare vascular anomaly characterized by arteriovenous shunting [2], and its presence in the IVC is particularly unusual. Gross examination of the excised specimen revealed an elongated pink tissue measuring 18 cm in length by 1.3 cm in diameter, further distinguishing it from typical thrombotic material. Management challenges in this case included procedural complications such as failure of the Inari ClotTriever catheter to retrieve the lesion, necessitating alternative approaches for removal. Additionally, given the patient’s history of intracranial hemorrhage, the use of thrombolytics required careful risk assessment and close monitoring in the ICU.

## Conclusions

This case underscores the importance of considering alternative diagnoses when evaluating IVC thrombus, particularly when imaging findings are atypical or when thrombolysis and thrombectomy fail. The diagnosis of AVH highlights the role of histopathological analysis in differentiating benign vascular lesions from thrombotic or neoplastic processes. A systematic diagnostic approach, multimodal imaging, and multidisciplinary collaboration were crucial in achieving an accurate diagnosis and appropriate management. Future considerations include long-term follow-up to assess for potential recurrence or hemodynamic consequences related to the resection of this rare vascular anomaly.
